# Projected distribution and climate refugia of endangered Kashmir musk deer *Moschus cupreus* in greater Himalaya, South Asia

**DOI:** 10.1038/s41598-020-58111-6

**Published:** 2020-01-30

**Authors:** Paras Bikram Singh, Kumar Mainali, Zhigang Jiang, Arjun Thapa, Naresh Subedi, Muhammad Naeem Awan, Orus Ilyas, Himal Luitel, Zhixin Zhou, Huijian Hu

**Affiliations:** 1 Guangdong Key Laboratory of Animal Conservation and Resource Utilization, Guangdong Institute of Applied Biological Resources, Xin’ganxi Road, Guangzhou, China; 2 National Socio-Environmental Synthesis Center, Annapolis, Maryland USA; 3 Key Laboratory of Animal Ecology and Conservation Biology, Institute of Zoology, Chinese Academy of Sciences, Beichen West Road, Beijing, 100101 China; 4 University of Chinese Academy of Science, Beijing, 100049 China; 5 Small Mammals Conservation and Research Foundation, Kathmandu, Nepal; 6 National Trust for Nature Conservation, Khumaltar, Lalitpur Nepal; 7 Earth Day Network, Islamabad, Pakistan; 8 Department of Wildlife Sciences, Aligarh Muslim University, Aligarh, India; 9 Center for Biotechnology, Agriculture and Forestry University, Rampur, Chitwan Nepal; 10 Conservation Innovation Center, Chesapeake Conservancy, Annapolis, Maryland USA

**Keywords:** Ecological modelling, Projection and prediction

## Abstract

Kashmir musk deer *Moschus cupreus* (KMD) are the least studied species of musk deer. We compiled genetically validated occurrence records of KMD to construct species distribution models using Maximum Entropy. We show that the distribution of KMD is limited between central Nepal on the east and north-east Afghanistan on the west and is primarily determined by precipitation of driest quarter, annual mean temperature, water vapor, and precipitation during the coldest quarter. Precipitation being the most influential determinant of distribution suggests the importance of pre-monsoon moisture for growth of the dominant vegetation, Himalayan birch *Betula utilis* and Himalayan fir *Abies spectabilis*, in KMD’s preferred forests. All four Representative Concentration Pathway Scenarios result an expansion of suitable habitat in Uttarakhand, India, west Nepal and their associated areas in China in 2050s and 2070s but a dramatic loss of suitable habitat elsewhere (Kashmir region and Pakistan-Afghanistan border). About 1/4^th^ of the current habitat will remain as climate refugia in future. Since the existing network of protected areas will only include a tiny fraction (4%) of the climatic refugia of KMD, the fate of the species will be determined by the interplay of more urgent short-term forces of poaching and habitat degradation and long-term forces of climate change.

## Introduction

The distribution of species around the world is not uniform; climate plays a vital role in defining species’ distributions and generating overall patterns of biodiversity in space and time. Species survive in a particular habitat mostly because they maintain an equilibrium with climate in their range^1–3^. Maintaining this equilibrium in the face of changing climate, however, may require a continuous shift in distribution. As the climate of the Himalaya is changing rapidly^4^, we expect species to adjust their distributions. Unusual patterns of precipitation and temperature have already been observed in the Himalaya and such patterns are predicted to become more severe in the future^5–7^. Actual and forecasted effects of climate change on biodiversity are expected to include: changes in phenology, tree line shifts, alien species invasions, declines in population and diversity, habitat alternations, and extinction^8–16^. Such effects could push populations of endangered species up to and beyond the verge of extinction. Hence, to maintain species diversity it is crucial to understand the impact of climate change on the distribution and habitats of endangered species, especially in the understudied areas like the Himalaya, which is already experiencing climate changes more rapidly than most of the planet is^17–22^.

Seven species of Musk deer *Moschus* spp. are endemic to the mountains of Asia and six of these are listed by the International Union for the Conservation of Nature and Natural Resources (IUCN) as endangered. Four of the endangered deers, i.e., the Kashmir musk deer *Moschus cupreus*, Himalayan musk deer *Moschus leucogaster*, Alpine musk deer *Moschus chrysogaster* and Black musk deer *Moschus fuscus*, inhabit the high Himalaya^23–25^. Poaching for musk and habitat fragmentation are the two major drivers of musk deer population decline^26,27^. Musk deer are habitat specialist, living in dense and undisturbed vegetation found above 2500 m^28–32^. Musk deer are a special case of convergence of challenges: they are endangered, they are habitat specialist^30^, and that they live in a montane system that is experiencing a rapidly changing climate^33,34^. Hence, it is crucially important to understand their current and future distribution if we are to keep them from going extinct. The understanding gained in examining these deer may also help us to understand the response and vulnerability of other montane species to the changing climate.

**Figure 1:**
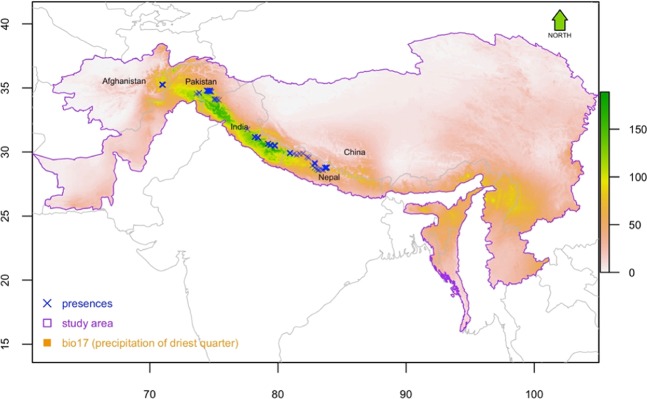
Occurrences (n = 136) of Kashmir musk deer against the backdrop of precipitation of the driest quarter, which was identified by this study as the most important predictor of musk deer distribution. A total of 123 points were obtained from 10 protected areas situated between central Nepal and Afghanistan through primary data collection efforts between 2003 and 2016. These data were supplemented with 13 points from Afghanistan and Kashmir region obtained from published sources. The map was plotted using R 3.4.3 (R Foundation for Statistical Computing, Vienna, Austria, https:// www.R-project.org).

Kashmir musk deer (*Moschus cupreus*, hereafter KMD) is the least studied among all species of musk deer of the Himalaya because it has historically been reported only from Kashmir and associated regions of India, Pakistan and Afghanistan (Fig. 1). These areas have experienced an extended period of intense armed conflict, making research expeditions highly perilous. KMD has been described only from the Kashmir region of the western Himalaya within the altitudinal range between 2000 and 4200 m asl. Very recent studies, however, indicated that the range of the species is much wider than previously thought. Ostrowski *et al*.^23^ confirmed the presence of KMD in Nuristan, northeast Afghanistan, which is the western limit of the species. In 2019, Singh *et al*.^35^ confirmed through genetic analyses that Kashmir musk deer populations occur in the Mustang area of central Nepal, whereas the deer populations to the east of Mustang, i.e. east of Annapurna Range in the Himalaya, are Himalayan musk deer *Moschus leucogaster*. Hence, Mustang is the eastern limit of KMD.

Pelage color of an individual can change with the seasons and similar kinds of variation in pelage color can be found within as well as between species^35–37^ making pelage color an unreliable metric for species identification. The misidentification of various species resulting in a mismatch between actual and perceived range, the lack of confirmatory occurrence records, and the non-rigorous data collection scheme based on opportunistic sightings calls into question the validity of previous species distribution models. For example, two studies (Khadka *et al*., 2017, Khadka and James 2017) predicted the suitable habitat of Himalayan musk deer in vast stretches across the Himalaya, from east to the west and from south to the north. These results are completely unrealistic as Himalayan musk deer exist only in the eastern Himalaya, whereas Kashmir musk deer are found in the western Himalaya^35,38,39^. Another study Lamsal *et al*.^40^ purported to analyze the distribution of Alpine musk deer, for the entire Himalaya of Nepal, but Alpine musk deer are restricted to central China^35,41^. Given the misidentification of species and incorrect occurrence records in the prior studies (Khadka *et al*., 2017, Khadka and James 2017, Lamsal *et al*., 2018), it is apparent that the distribution of musk deer in the Himalaya needs to be reexamined.

In contrast to these prior studies, we did a number of things to ensure our occurrence records and habitat analyses produced high quality data. First, we used recent genetic analysis^35,38,39^ to confirm the populations of Kashmir musk deer and Himalayan musk deer in southern parts of the greater Himalaya. Second, we obtained distributional data of KMD by systematically surveying five protected areas in Nepal, three in India, and one in Pakistan between 2003 and 2017. Third, we searched for the locations of musk deer latrines which are set up only in high quality habitat. The deer uses latrine sites regularly throughout the year for defecation, for establishing communications with conspecifics, and to maintain territory^35,42^. Based on their unique location and repeated use, we suggest that latrine sites represent high quality habitat sites for musk deer, which is in contrast with opportunistic sightings (which can capture low quality habitat in the data) and unverified locations (when analysis is based on occurrence records maintained by public domain like GBIF).

In this study, we modelled the distribution of the least studied endangered Kashmir musk deer with the following objectives: (1) understand the current and expected future distribution of the species under different climatic scenarios, (2) identify potential climatic refugia for the species, and (3) determine the adequacy of the existing network of protected areas for protecting this species in future.

## Results

### Important predictors of species distribution

**Figure 2:**
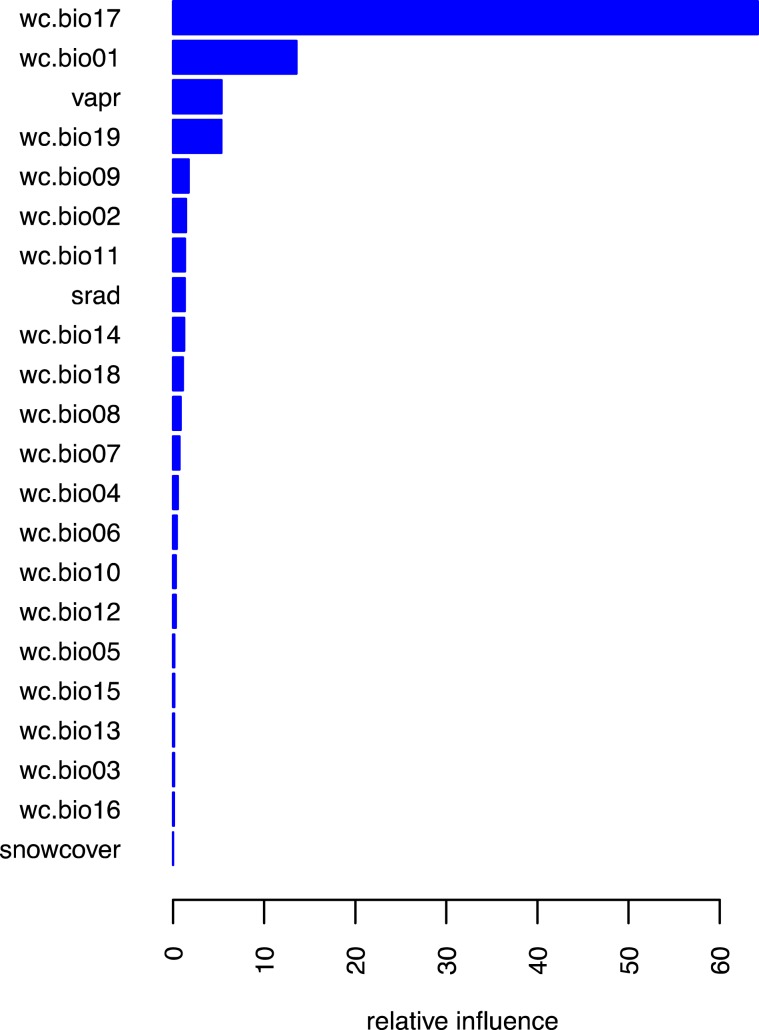
The relative influence of the predictors in Kashmir musk deer distribution; predictors are listed on y-axis. We started our model construction process with 26 potential predictors (Table 1). However, four of these covariates (land cover, aspect, altitude and distance to the nearest water source) were not available for future climate scenarios at our desired spatial resolution or required enormous computational resources. These covariates were some of the least important predictors (relative influence <3), and therefore were dropped from further analysis. The remaining 22 predictors (Table 1) were used to develop the model.

Out of 22 selected covariates, we selected for species distribution modeling, four were found to have disproportionately high influence in determining the distribution of the KMD (Fig. 2). These include precipitation of the driest quarter “bio17” (relative influence = 65%) and annual mean temperature “bio1” (relative influence = 18%), as the two top most predictors. Water vapor “vapr” and precipitation during coldest quarter “bio19” played a minor but still important role in species distribution. Collectively, the predictors possessed relative influence equal to 93% in model performance.

### Projected distribution under current climatic conditions

**Figure 3:**
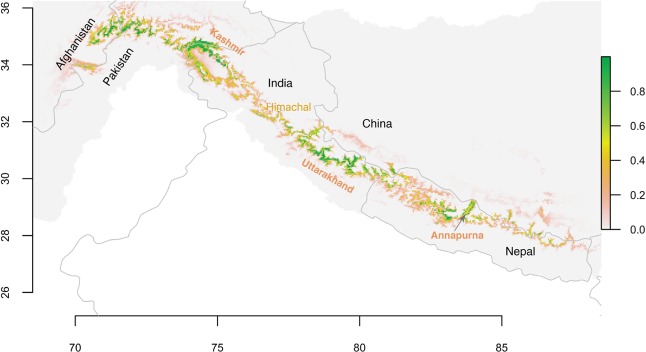
Habitat suitability is displayed as a continuous quantity between zero and one. The probability surface was generated by species distribution models built with Maximum Entropy (MaxEnt) Models using R 3.4.3 (R Foundation for Statistical Computing, Vienna, Austria, https:// www.R-project.org).

The prediction of the species distribution models yielded probability of occurrence or habitat suitability (Fig. 3). KMD is predicted to inhabit a belt of high Himalaya that stretches from central Nepal to north-west of India, reaching Afghanistan through the Kashmir Region of India and Pakistan. However, the suitable habitats do not occur in a continuum throughout the high Himalaya. Suitable habitats are located west of Annapurna Himalaya range including Mustang (hereafter Annapurna region), west of Annapurna region (hereafter, west Nepal), the northern part of Uttarakhand state of India (hereafter, Uttarakhand), west of Uttarakhand in Himachal Pradesh (hereafter Himachal), the Kashmir region of India and Pakistan (hereafter, Kashmir region), and along and close to the northeast border between Pakistan and Afghanistan (hereafter, Pak-Afghan border). Additionally, small patch of suitable habitat observed in Lubu area in China nearby north west border between Nepal and China. Few patches of suitable habitat occur in the the eastern half of Nepal and their adjacent area in Tibet as this area supports the closely related Himalayan musk deer.

When the continuous probability surface was classified into categories, we observed that highly suitable (probability >0.7) and suitable (probability 0.5–0.7) habitats are distributed in patches throughout the Himalaya from central Nepal to north east Afghanistan (Supplementary Fig. S1). The continuous patches of high quality habitat are located in Pak-Afghan border, Kashmir region, Uttarakhand and in Annapurna region. In other regions (Himachal, west Nepal, west of Kashmir region) suitable habitats are presented in different fragments. The areas where suitable and highly suitable habitats are presented also include marginally suitable habitat (0.2–0.5) west of Annapurna to Pak-Afghan border whereas high proportion of marginally suitable habitat are observed east of Annapurna where closely related Himalayan musk deer are present.

### Projected distribution of KMD under predicted future climate scenarios

**Figure 4:**
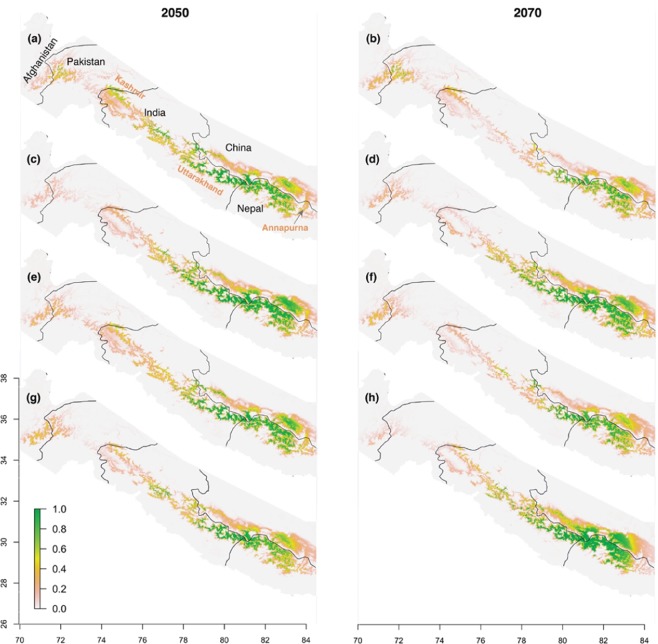
Estimated habitat suitability in 2050 and 2070 under various climate change scenarios. The prediction was made for the entire Himalaya (see Fig. 1), but we only show the part that includes all sites with suitable habitat. (**a**) RCP 2.6 climate scenario in 2050s. (**b**) RCP 2.6 climate scenario in 2070s (**c**) RCP 4.5 in 2050s0s. (**d**) RCP 4.5 in 2070s. (**e**) RCP 6.0 in 2050s. (**f**) RCP 6.0 in 2070s. (**g**) RCP 8.5 in 2050s. (**h**) RCP 8.5 in 2070s. All maps were plotted using R 3.4.3 (R Foundation for Statistical Computing, Vienna, Austria, https://www.R-project.org).

When predicted distribution under future climate change scenarios are examined collectively, northwards shifting and disappearance of habitat were observed. In west Nepal and Uttarakhand, the habitat shifts northward, resulting a continuous distribution of highly suitable habitats in north part of both areas (west Nepal and Uttarakhand) and their adjacent areas in Tibet in future in all four RCP scenarios in 2050s and 2070s. However, the extent of suitable habitat will differ in four scenarios. In 2050s (RCP 2.6, RCP 4.5, RCP 6.0 and RCP 8.5), most of the suitable habitat will be available in west Nepal and Uttarakhand as well as their adjacent area in Tibet along the border between three countries (Nepal, India and China). In RCP 2.6 scenario in 2050, some patches of suitable habitat will appear in Himachal between Kashmir region and Uttarakhand in future whereas most of the habitat will disappear from Kashmir region and Pak-Afghan border. In RCP 4.5, RCP 6.0 and RCP 8.5 scenarios, by 2050, KMD’s suitable habitat will disappear from Kashmir region, Pak-Afghan border and Annapurna region. In all four scenarios during 2070s, the suitable habitat from Pak-Afghan border, Kashmir region and Himachal region will disappear. In the rest of Himalaya to the west of Uttarakhand (the Indian Himalaya, all Himalaya of Pakistan and Afghanistan Himalaya), currently observed suitable and highly suitable habitats will be entirely lost in future (2050s and 2070s) in three scenarios (RCP 45, RCP 60 and RCP 85). In the Pak-Afghan border, Pakistan, Kashmir region, and central Nepal regions only marginally suitable are predicted to be present in future during 2050s and 2070s (Fig. 4).

Compared to the other RCP scenarios (RCP 2.6, RCP 6.0 and RCP 8.5), by 2050 the habitat for KMD under the RCP 4.5 will expand more and the habitat of this deer is expected to be distributed densely within west Nepal, Uttarakhand and their associated areas in Tibet (Fig. 4). By 2070, habitat in RCP 4.5 and RCP 8.5 scenarios will depict similar pattern (Fig. 4). However, the habitat of KMD will be expanded in Uttarakhand, west Nepal and their associated areas in Tibet in RCP 8.5 scenarios (Fig. 4). The habitat in RCP 2.6 in 2070s will be found sparsely in east of Uttarakhand, west of west Nepal and their adjacent area in Tibet compare to other three scenarios. Habitat of KMD in three scenarios (RCP 26, RCP4.5 and RCP 6.0) will decrease in 2070s compare to 2050s in Uttarakhand, west Nepal and their associated areas in Tibet. However, habitat of KMD in RCP 8.5 will increase in 2070s compare to 2050s (Fig. 4).

In future, HSH (Highly Suitable Habitat) for KMD will dominate over SH (Suitable Habitat) and MSH (Marginally Suitable Habitat) in Uttarakhand and west Nepal in all four climatic scenarios. In Tibet, HSH dominate MSH and SH in three cases i.e. RCP 4.5 and RCP 6.0 in 2050s, RCP 4.5 and RCP 8.5 in 2070s whereas SH and MSH dominate HSH in five cases i.e. RCP 2.6, RCP 6.0 and RCP 8.5 in 2050, RCP 2.6, RCP 4.5, RCP 6.0. Some patches of SH will remain in Pak-Afghan border, Kashmir region and Himachal area in RCP 2.6 scenario in 2050s. In other scenarios, only MSH will exist in Pak-Afghan, Kashmir and Annapurna regions where some patches of HSH and HS will be available in Himachal areas except in RCP 2.6 in 2070s (Supplementary Fig. S2).

**Figure 5:**
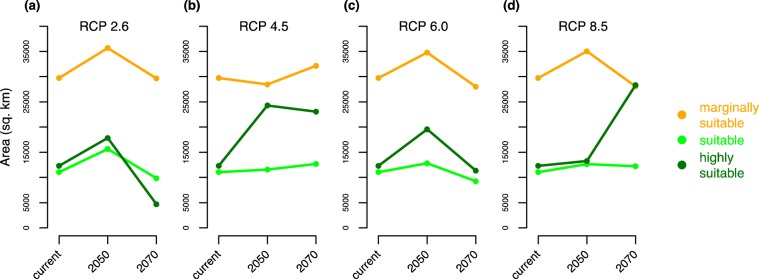
Change in area of suitable habitats in future. The total area of all three types of suitable habitats was computed for all climate scenarios: RCP 2.6 (**a**), RCP 4.5 (**b**), RCP 6.0 (**c**) and RCP 8.5 (**d**). The area each grid cell was calculated separately from a raster in the Geographic Coordinate System accounting for Earth’s curvature, rather than in a flat Cartesian coordinate system.

When the entire Himalaya was examined collectively, we observed that the HSH and SH will increase in all scenarios (RCP 2.6, RCP 6.0, RCP 4.5 and RCP 8.5) during 2050s whereas HSH and SH will increase than now in two scenarios (RCP 4.5 and RCP 8.5) during 2070s and decrease in remaining scenarios (RCP 2.6 and RCP 8.5) (Fig. 5). Except in 8.5 scenario in 2070s, HSH and SH will diminish than 2050s. MSH will maintain similar pattern between now and 2070s in all scenarios whereas MSH will increase in three scenarios (RCP 2.6, RCP 6.0 and RCP 8.5) in 2050s. Area of MSH will decrease in 2070s than 2050s in all scenarios except RCP 4.5. More habitat is expected to be available for KMD in Uttarakhand, west Nepal and their associated areas in Tibet in future. Both HSH and SH will dominate Uttarakhand, west Nepal and Tibet in 2050s and 2070s.

### Protected areas and refugia

**Figure 6:**
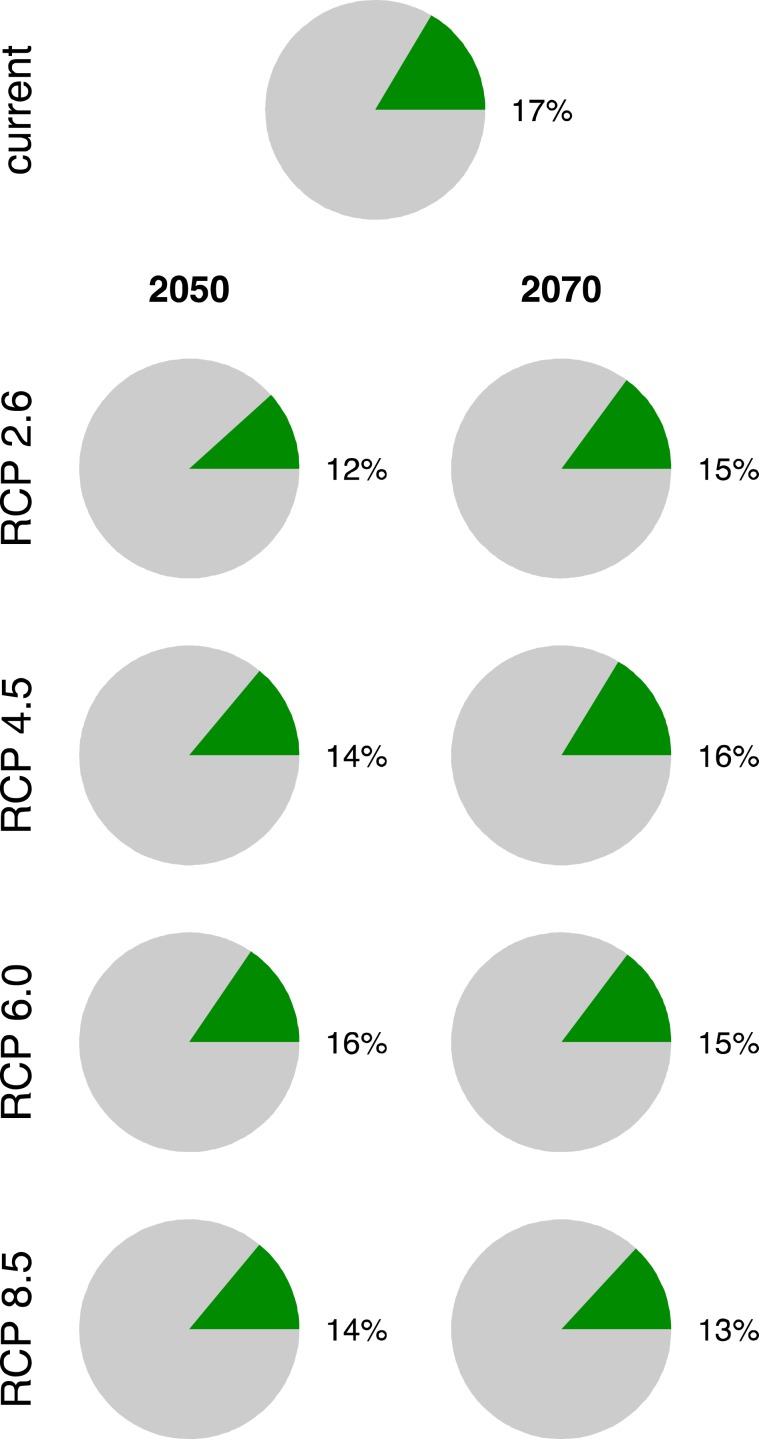
Fraction of the projected species range that is inside currently designated protected areas. The species range is the totality of grid cells that have the probability of at least 50% suitable, and highly suitable sites. This fraction was computed as the area of the range inside a protected areas divided by the total range area. The Earth’s curvature was accounted for determining the area with the function raster:area() in R 3.4.3 (R Foundation for Statistical Computing, Vienna, Austria, https://www.R-project.org).

**Figure 7:**
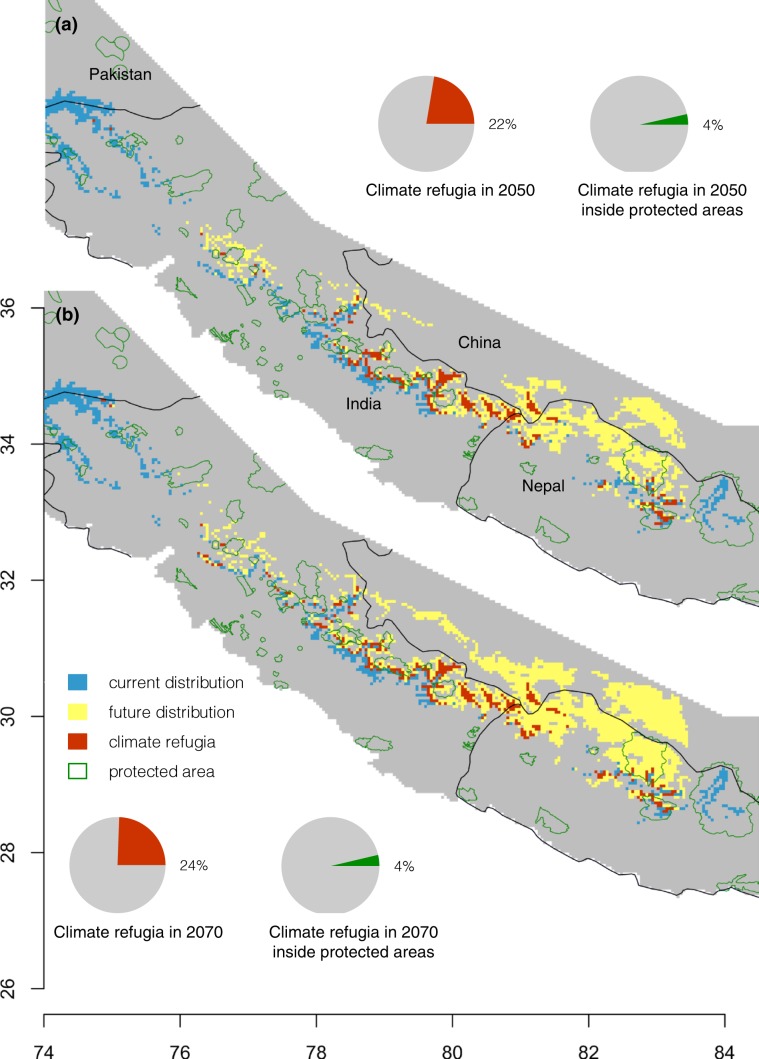
Climate refugia and protected areas. Climate refugia are assumed to occur where the current species range overlaps with the predicted future range. Spatial distributions of climate refugia for 2050s (**a**) and for 2070s (**b**) under RCP 8.5 are shown. Next to each of these maps are two pie charts that display what fraction of the species current range remains as a potential climate refugia (red pie) and the fraction of that climate refugia that is located inside protected areas (green pie). All maps were plotted using R 3.4.3 (R Foundation for Statistical Computing, Vienna, Austria, https://www.R-project.org).

A small fraction of KMDs range now or likely in the future (Fig. 6) are being protected by the currently designated network of protected areas (green shade in Fig. 7). Currently, only 17% of range inside the protected areas contains habitat with 0.5 or greater suitability. Many of the protected areas are located north of KMD’s currently suitable habitat. As suitable habitats shift northward, more of the future range will fall outside currently protected areas (12–16%). If a site provides current and predicted future suitability, it carries higher conservation value because it could serve the purpose of a climate refugia potentially allowing that local population of KMD from needing to migrate to survive. By overlapping predicted future species range maps with the current ranges, we show that 22% and 24% of current species range will serve as climate refugia in 2050 and 2070, respectively (Fig. 7). Unfortunately, only 4% of these potential refugia are located inside present protected areas.

## Discussion

### Distribution of KMD: A game of moistures

The growth limiting factor for the species of trees in KMD (Kashmir Musk Deer) habitat is the moisture available during the pre-monsoon season^43–47^. In the southern Himalaya, moisture comes from precipitation arising from the Indian Ocean mainly during summer, making high rainfall in the eastern Himalaya leaving the western Himalaya drier than eastern Himalaya. During winter, the westerly winds from the Mediterranean sea brings more precipitation in the form of snow to the western Himalaya, but less to the eastern Himalaya^48^. The massive mountain range acts as a barrier to air flow from south to north, resulting in a dry and treeless Tibet^49^. Most of the coniferous trees respond negatively with temperature when temperature increase above 6.6 °C and positively with precipitation during pre-monsoon^50–52^. Spruce *Picea smithiana*, fir *Abies pindrow*, yew *Taxus wallichiana*, bluepine *Pinus wallichiana*, Himalayan birch *Betula utilis* and rhododendron *Rhododendron campanulatum* etc. are commonly recorded tree in the habitat of KMD. Growth of these trees are limited by moisture in pre-monsoon^43–45,53^. Our finding also suggests that Himalayan trees are more responsive to changes in the regime of precipitation than temperature. The strong influence of precipitation to KMD distribution likely results from the sensitivity of forests supporting KMD sites to adequate rainfall. The distributional boundaries we predicted are echoed by recent studies: Singh *et al*.^35^ indicated Mustang, central Nepal as the eastern boundary and Ostrowski *et al*.^23^ indicated Nuristan, Afghanistan as the western boundary. However, the distributional range of KMD as depicted by IUCN is markedly different from our data-driven modeling studies IUCN^32,35^.

### Future distribution of KMD

West Nepal, Uttarakhand, Kashmir region, and Pak-Afghan border will support highly suitable and suitable habitat. Most of the current habitat of KMD will disappear in the 2050s or the 2070s in all climatic scenarios (RCP 2.6, RCP 4.5, RCP 6.0 and RCP 8.5) except Uttarakhand and west Nepal and their adjacent areas in Tibet. However, few patches of suitable habitat will remain in Himachal and close to Pak-Afghan border in 2050s in RCP 2.6 scenario. Mean temperature is predicted to rise by 1–2 °C in 2050s and by 1–3 °C in 2070s in four RCP scenarios^54^. Although temperature will keep rising, precipitation is predicted to behave differently depending on the amount of moisture in the air, where it comes from, and the convergence of air currents^55,56^. In the range of KMD, sites that are unsuitable today to the north of Uttarakhand and western Nepal will receive more pre-monsoon moisture in future (see Supplementary Note). This explains why those currently uninhabited areas show a suitable sites in future climates (Fig. 4).

Himalayas in Uttarakhand and west Nepal are not higher as Himalayas in Annapurna region, Pakistan and Kashmir region. Mountains above 8000 m from mean sea level are located either in Pakistan or Annapurna region and east of it. Not surprisingly this huge area and its diverse topography manifests in a very complex climate system that is not well understood^57^. This distinct dynamism of temperature and precipitation due to geographical differences between the western regions (Kashmir and Pak-Afghan) and the eastern region (Uttarakhand and west Nepal) are enough to justify the reason behind loss of habitat of KMD west of Uttarakhand (Kashmir region, Afghanistan and Pakistan) in future (2050s and 2070s).

Contrary to our finding, Khadka *et al*., 2017 found temperature play a greater role in distribution of musk deer. Therefore, Khadka *et al*.^58^ predicted (i) that vast stretches of currently suitable habitat of Himalayan musk deer exist at very high elevation including above tree line and snowline although we know that the species needs a forest to survive, and (2) that this suitable habitat expands dramatically in future to higher elevation areas without any contraction. The unrealistic future projection of musk deer by Khadka *et al*., 2017 has another layer of complexity: plants do not respond to newly available suitable climate above treeline and snowline because soil may not have formed in enough quantity to support the plant growth in such large area^59,60^. Collectively, these lines of arguments and evidences support our finding that current species range of KMD will respond to future climate in a complex fashion, which is different from a species that is primarily controlled by temperature. We show that all kinds of habitats in Kashmir region, Himachal and Pak-Afghan border will be the ones showing the first sign of climate change; they will decline in the 2050s and 2070s.

There are a few reasons why prior modeling studies (e.g., Khadka *et al*. 2017, Khadka and James 2017, Lamsal *et al*. 2018) resulted in unrealistic projection of species distribution, and they relate to the way they identified species. Pelage color is not a reliable metric for identifying species of musk deer. Pelage color may vary within a species and it changes with season. Different species can look alike as well as individuals of the same might look different^35^. Fecal pellets are not uniquely identified at a species level. Occurrences based on opportunistic sightings and anecdotal information has the potential to hamper the model as such occurrences have a higher chance of being incorrect or falling in lower quality habitat than our systematic survey of latrines. Khadka *et al*., 2017 and Khadka and James 2017 concluded that temperature (relative importance = 74%) is the most important variable influencing Himalayan musk deer distribution. In water scarce environments, a small difference in moisture availability can make a difference in habitat quality. Such ecological expectations can also be inferred from the fact that forest cover is an important factor to KMD given their timid nature^30^ and vegetation growth is largely influenced by moisture in dry environments^60–64^. These deer occur sparsely if there is inadequate dense vegetation cover (>40% crown cover) to hide within during the day^30^.

### Protected, non-protected habitats and climate refugia

We observed that only 17% of the current suitable range (projected probability >0.5) falls inside protected areas (PAs). In the future, this will decrease to 12–16%. The range are in low fraction and other researchers have reported similarly low fraction of range protected inside conservation area for other species of Himalayan range such as 19% for snow leopard^61^. Large portions of the suitable habitat of musk deer are located outside PAs where anthropogenic pressure are highly threatening biodiversity^65,66^. Only 4% of habitat in 2050s and 2% of habitat in 2070s will act as climate refugia inside PAs. However, 25% of current suitable range in 2050s and 16% in 2070s will act as climate refugia in entire range of KMD.

### Climate refugia conservation; next generation conservation

Musk deer evolved 4 million years ago in Tibetan plateau^67^ and survived until now despite various climate changes during the Quaternary period as well as after remarkable geological process in Asia including upliftment of Himalaya^68,69^. Climatic refugia are a plausible explanation for its survival through different climate changes, allowing the species later to disperse in the mountains of Asia when situation slowly normalized. Another plausible explanation for the survival relates to the ability of a species to cope with the changing environment and its interaction with other species. Unfortunately, the velocity of the climate change is faster than the response of the species is^70^. Therefore, the role of climate refugia is always crucial. The extinction of megafauna, such as woolly mammoth *Mammuthus primigenius* and Giant deer *Megaloceros giganteus* during the late Quaternary period was mainly because they could not find such refugia to survive through the glacial-interglacial cycle^71^. In the case of KMD, about quarter of the current range can serve as climate refugia in future (2050s and 2070s). These refugia are situated in Uttarakhand, west Nepal and west of Annapurna region.

## Material and Methods

### Study area: The greater himalaya

The greater Himalaya are often called the roof of the world and they extend from northern Pakistan to north east India throughout Nepal, Bhutan and the southern part of Tibet. Western disturbances from the west arise from the Mediterranean Sea and dominate the climate of western Himalaya during winter. During summer, the dominant weather pattern is driven by moisture arising from the Indian Ocean^48,72,73^. In Uttarakhand and west Nepal, the climate is different than Kashmir and Pak-Afghan as it is influenced by both south west monsoon and westerly storms^74^. The different weather phenomena in western and eastern Himalaya has resulted in a drier west and wetter east^75^. Hence, species composition are different in both regions of Himalaya. Western Himalaya temperate forests exhibits both broadleaf and conifer forest where spruce *Picea smithiana*, fir *Abies pindrow*, yew *Taxus wallichiana*, bluepine *Pinus wallichiana* and rhododendron *Rhododendron campanulatum* are common species. In eastern Himalaya, eastern Himalayan broadleaf forests, eastern Himalaya subalpine conifer foersts, northern trinagle temperate forests and northeastern Himalayan subalpine conifer forests form temperate forest^76–78^. Western tragopan *Trogopan melanocephalus*, cheer pheasant *Catreus wallichii*, Kashmir musk deer *M.cupreus*, Markhor *Capra falconeri* etc. are only found in western Himalaya whereas Alpine musk deer *M. chrysogaster*, Himalayan musk deer *M. leucogaster*, black musk deer *M. fuscus*, red panda *Ailurus fulgen*, Bhutan Takin *Budorcas taxicolar whitei* etc. are recorded only in eastern Himalaya.

### Presence records of musk deer

Following the genetic study of the samples collected in the Nuristan region of Afghanistan and the Mustang region of Annapurna Himalaya range in Nepal (Singh *et al*., 2019), Nuristan is confirmed as the western limit and Mustang as as the eastern limit of the KMD range. We determined the geographic coordinates of latrines between these two distribution limits of KMD in South Asia. We documented 136 occurrence records of KMD, 123 of them being in our primary collection area within five protected areas in Nepal (Annapurna Conservation Area, Dhorpatan Hunting Reserve, Shey Phoksundo National Park; Rara National Park, Api Nampa Conservation area), Musk Deer National Park in Pakistan, Nuristan of Afghanistan, and four protected areas in India (Dachigam National Park, Kedarnath Wildlife Sanctuary, Nanda Devi Biosphere, Govinda Pashu Vihar Wildlife Sanctuary). In total, we collected 45 latrine samples in Nepal, 61 in India, 21 in Pakistan, and 9 in Afghanistan (Fig. 1).

Latrines, which serves as an indisputable evidence of deer presence, can easily be distinguished from the excreta of other animals; they have the heaps of old and fresh pellets with a musky smell^42,79–81^, and the pellets are much smaller and cylindrical compared to that of goat *Capra aegagrus* and sheep *Ovis aries*. Musk deer are shy, nocturnal and crepuscular forest-dweller species^82^. During the day, they hide in vegetation. Hence, musk deer are mostly inconspicuous to human. However, musk deer develop latrines by defecating repeatedly at a single site to maintain communication for various proposes^81,83,84^.

Geographical locations of latrine sites were determined first in Mustang, Nepal. In these expeditions, we performed systematic surveys for understanding habitat by laying out quadrats of size 20 m × 20 m along transects at different elevations. In the summer and autumn of 2016–2017, we visited several areas up to 4500 m in the high Himalayans of Nepal (Manang, Mustang, Kaski, Bhimthang of Annapurna region). Occurrence records from remaining four protected areas of Nepal were collected by random sightings of latrine sites and animals by the game scouts and rangers working in each protected area. For India and Pakistan, we used a retrospective study design. Coordinates of latrine sites systematically collected for Alpine musk deer from Uttarakhand, India and from Pakistan by co-author of this paper from 2003 to 2017 were considered to be KMD instead of Alpine musk deer. Data in Pakistan were collected from systematically distributed quadrats. Additionally, published literature was reviewed and presence locations of musk deer from Afghanistan and Dachigam National Park, Kashmir region, India were extracted by scanning and georeferencing previously produced range maps (Fig. 1). The data obtained from previously published sources (secondary data) comprised of less than 10% of our pool of occurrence records whereas our primary data collection effort contributed over 90% of the occurrence records.

### Potential predictors

The current climate is represented by raster layers for 19 climatic variables with the resolution of 30 arc seconds (approximately 1 km) (Table 1) obtained from WorldClim version 2.0^85^. These data were fitted to the geographic boundaries of the Hindu Kush Himalaya as defined by the International Centre for Integrated Mountain Development (ICIMOD). Based on our understanding of the distribution and ecology of KMD, we supplemented this standard set of potential covariates with additional relevant variables (Table 1). In total, 26 covariates were applied to the first set of models.

**Table 1:** Potential predictors for Kashmir musk deer distribution.

S. N.	Potential covariates	Data format	Source
1	wc.bio1	Annual Mean Temperature	WorldClim 2.0
2	wc.bio2	Mean Diurnal Range	WorldClim 2.0
3	wc.bio3	Isothermally	WorldClim 2.0
4	wc.bio4	Temperature Seasonality	WorldClim 2.0
5	wc.bio5	Max Temperature of Warmest Month	WorldClim 2.0
6	wc.bio6	Min Temperature of Coldest Month	WorldClim 2.0
7	wc.bio7	Temperature Annual Range	WorldClim 2.0
8	wc.bio8	Mean Temperature of Wettest Quarter	WorldClim 2.0
9	wc.bio9	Mean Temperature of Driest Quarter	WorldClim 2.0
10	wc.bio10	Mean Temperature of Warmest Quarter	WorldClim 2.0
11	wc.bio11	Mean Temperature of Coldest Quarter	WorldClim 2.0
12	wc.bio12	Annual precipitation	WorldClim 2.0
13	wc.bio13	Precipitation of Wettest Month	WorldClim 2.0
14	wc.bio14	Precipitation of Dries Month	WorldClim 2.0
15	wc.bio15	Precipitation Seasonality	WorldClim 2.0
16	wc.bio16	Precipitation of Wettest Quarter	WorldClim 2.0
17	wc.bio17	Precipitation of Driest Quarter	WorldClim 2.0
18	wc.bio18	Precipitation of Warmest Quarter	WorldClim 2.0
19	wc.bio19	Precipitation of Coldest Quarter	WorldClim 2.0
20	srad	Solar radiation	WorldClim 2.0
21	vapr	Water vapor	WorldClim 2.0
22	snowcover	Snow cover	WorldClim 2.0

We initially incorporated 26 covariates in species distribution model. Four of these, i.e., land cover, aspect, altitude, distance to water bodies were dropped from further analyses because of their static nature. The final model analyses included the 22 covariates listed below.

Our initial modeling exercise indicated that several of these covariates secured a very low variable importance. Some of these minimally important covariates also had other problems. Specifically, “distance to the nearest source of water” required computing distance from each of our occurrences and background points to the water bodies and identifying the nearest source. This turned out to be computationally intensive. So, we safely dropped minimally important covariates “distance to the nearest source of water”. Further, we dropped “aspect” “altitude” and “land cover” because of their static nature and they could lead to over fitting the model. We also adapted filter i.e. approach to eliminate over fitting. This approach was useful in optimizing the model coefficients such that a more stable model is trained. Specifically, we tested spatial transferability of the models by comparing various model evaluation metrics^86–88^. The goal here is to find a model that performs in both training and testing regions similarly. With various regularization multipliers, we found that a value of 2 minimizes the delta AIC (difference in AIC of the models in training vs testing regions). Finally, we selected 22 variables for processing our model to understand present distribution of the KMD. To forecast range patterns in the years 2050 or 2070, we used the same variables examined the present distribution of KMD in this study and applied the four climate change scenarios as used in Fifth Assessment by the International Panel on Climate Change (Representative Concentration Pathways RCP2.6, RCP4.5, RCP6, and RCP8.5). For forecasting future distribution, we used Global Circulation Model (GCM). We reviewed various literatures related to GCM. BCC-CSM1-1, CCSM4, HadGEM2-CC and MIROC 5 have been mostly used in SDM to perform future prediction. All of these models are also used in Fifth Assessment IPCC report (IPCC AR5). CMIP5 publications database reveals that CCSM4 is at top level which has been used in 440 articles, which is followed by MICROC 5 (409) and then by BCC-CCM1-1 (389). HadGEM2-CC has been used by 337 publications. We selected BCC-CSM1-1 because it is based on CCSM and itself widely used for projecting future distribution^89^. Therefore, using BCC-CSM1-1 provides double benefit over other GCMs. Other reasons behind applying BCC-CSM are i.) BCC-CSM1-1 is developed to issue global climate predictions and impact assessments at monthly, seasonal and inter-annual time scales, particularly over East Asiaand ii.) to do research on climate and climate change issue^90^. Our future predictions are related to climate change issues. We found BCC-CSM1-1 most fit in our case. All variables were resampled with the resolution of 30 arc seconds to use in model.

### Maximum entropy (MaxEnt)

MaxEnt is a widely used Species Distribution Model (SDM) for predicting species distribution in the fields of ecology and conservation^64,91–93^. MaxEnt^91^ operates by computing the ratio of the probability density function of multivariate environmental space of covariates in observed locations to that of the entire study area and it is particularly suitable for modeling presence-only data. It is mathematically related to Poisson Point Process^94^, another model suitable for presence-only analysis. Elith and Leathwick (2009) consider MaxEnt to be a particularly efficient model and we determined it was a good choice for modeling the distribution of KMD.

An important assumption of SDM is that distributional data of a species can be collected by either randomly or systematic sampling of occurrence records^95^. Our occurrence data were collected systematically in Annapurna Conservation Area (Annapurna region), Api Namba Conservation Area in Nepal, Nanda Devi Biosphere, Govinda Pashu Vihar Wildlife Sanctuary but the same scheme was not used in Afghanistan, Kashmir region and far western Nepal. Therefore, it can be argued that the occurrences might have some level of sampling bias. When background points are drawn with similar bias as presences, this, in theory, dampens the impact of sampling bias in presences. As expected, Phillips *et al*. 2009 showed improved model performance with biased background drawing. The bias can be directly obtained from the intensity of presences in the grid cells but making sure that the grid cells without any occurrences still contribute the background points with a much smaller probability^96^.

We sampled background points from occupied grid cells with a bias equal to the intensity of presences. For unoccupied grid cells, we sampled background points at two probabilities: 0.01 and 0.1, and compared the predicted surface and evaluation metrics of these three schemes of background drawing (two discussed above plus one without applying any bias in the entire study area). We checked current range of the species resulted from three types of biases with the precisely known habitat of the species in reference to Himalaya range. We found that the results slightly varied among the three types of biases (AUC = 0.99 for 0.1 bias, 0.96 for 0.01 bias and 0.99 without bias). We will report results only for 0.1 bias because this secured highest AUC score. Following Guo *et al*., 2017, we classified the continuous probability into four categories: unsuitable (0–0.2), marginally suitable (0.2–0.5)(MH), suitable (0.5–0.7)(SH), and highly suitable (0.7–1.0)(HSH)^60^ to understand current and future distribution of KMD.

### Analysis

All analysis and plotting were done with R 3.4.3^97^. The main analysis of SDM was performed with the packages “dismo” and “gbm”. The following packages were used in other analyses and plotting: “ENMeval”, “raster”, “sp”, “maps”, “rgeos”, “plyr”, “matrixStats”, “scales”.

## Supplementary information

Supplementary Notes.

Supplementary Figures.

## Data Availability

Musk deer is endangered species and poaching for the musk is major cause of population decline of the deer. Our presence records are location of latrine sites and it was found that poachers rely on freshness of latrines site to track the deer. These sites are used repeatedly by musk deer for many years. Therefore, we can not make our presence data publicly available. However, we can provide these data upon the formal request from the researchers.
