# Author Correction: Projected distribution and climate refugia of endangered Kashmir musk deer *Moschus cupreus* in greater Himalaya, South Asia

**DOI:** 10.1038/s41598-020-63413-w

**Published:** 2020-05-08

**Authors:** Paras Bikram Singh, Kumar Mainali, Zhigang Jiang, Arjun Thapa, Naresh Subedi, Muhammad Naeem Awan, Orus Ilyas, Himal Luitel, Zhixin Zhou, Huijian Hu

**Affiliations:** 1 Guangdong Key Laboratory of Animal Conservation and Resource Utilization, Guangdong Public Laboratory of Wild Animal Conservation and Utilization, Guangdong Institute of Applied Biological Resources, Guangzhou, China; 2 National Socio-Environmental Synthesis Center, Annapolis, Maryland USA; 3 Key Laboratory of Animal Ecology and Conservation Biology, Institute of Zoology, Chinese Academy of Sciences, Beichen West Road, Beijing, 100101 China; 4 University of Chinese Academy of Science, Beijing, 100049 China; 5 Small Mammals Conservation and Research Foundation, Kathmandu, Nepal; 6 National Trust for Nature Conservation, Khumaltar, Lalitpur Nepal; 7 Earth Day Network, Islamabad, Pakistan; 8 Department of Wildlife Sciences, Aligarh Muslim University, Aligarh, India; 9 Center for Biotechnology, Agriculture and Forestry University, Rampur, Chitwan Nepal; 10 Conservation Innovation Center, Chesapeake Conservancy, Annapolis, Maryland USA

Correction to: *Scientific Reports* 10.1038/s41598-020-58111-6, published online 30 January 2020

The original version of this Article contained an error in the Affiliation 1, which was incorrectly given as ‘Guangdong Key Laboratory of Animal Conservation and Resource Utilization, Guangdong Institute of Applied Biological Resources, Xin’ganxi Road, Guangzhou, China’ The correct affiliation is listed below:

Guangdong Key Laboratory of Animal Conservation and Resource Utilization, Guangdong Public Laboratory of Wild Animal Conservation and Utilization, Guangdong Institute of Applied Biological Resources, Guangzhou, China

Additionally, in the original version of the Supplementary Information file supplied with this Article an affiliation for Kumar Mainali was omitted.

These errors have now been corrected in the HTML and PDF versions of the Article and accompanying Supplementary Files.

