# Supplementary material for: Projected distribution and climate refugia of endangered Kashmir musk deer Moschus cupreus in greater Himalaya, South Asia
Source: Sci Rep. 2020 Jan 30;10:1511. doi: 10.1038/s41598-020-58111-6 (PMC6992763; doi:10.1038/s41598-020-58111-6)
Supplement: Supplementary file 1 — Supplementary Notes. [file 41598_2020_58111_MOESM1_ESM.docx]

# Projected distribution and climate refugia of endangered Kashmir musk deer *Moschus cupreus* in greater Himalaya, South Asia

Paras Bikram Singh ^1,6^, Kumar Mainali ^2^, Zhigang Jiang^3, 4^, Arjun Thapa ^5^, Naresh Subedi ^6^, Mahammad Naeem Awan ^7^, Orus Ilyas ^8^, Himal Luitel^9^, Zhixin Zhou^1^, Huijian Hu ^1*^

***^1^*** *Guangdong Key Laboratory of Animal Conservation and Resource Utilization, Guangdong Institute of Applied Biological Resources, Xin’ganxi Road, Guangzhou, China,* ***^2^****National Socio-Environmental Synthesis Center, Annapolis, Maryland, USA,* ***^3^****Key Laboratory of Animal Ecology and Conservation Biology, Institute of Zoology, Chinese Academy of Sciences, Beichen West Road, Beijing 100101, China,* ***^4^****University of Chinese Academy of Science, Beijing 100049, China, , ^5^Small Mammals Conservation and Research Foundation, Kathmandu, Nepal, ^6^* *National Trust for Nature Conservation, Khumaltar, Lalitpur, Nepal, ^7^Earth Day Network, Islamabad, Pakistan, ^8^Department of Wildlife Sciences, Aligarh Muslim University, Aligarh, India, ^9^Center of Biotechnology, Agriculture and Forestry University, Rampur, Chitwan, Nepal.*

*Corresponding author, email:13922339577@139.com

**Spatial Expansion of Suitable Level of bio17 for KMD**

Bio17 is the most important predictor of KMD distribution. The response curve shows that Bio17 makes an inflection around at bio17 = 75 and probability = 0.5. In the bio17 raster of current and future climates, we saved only those pixels with bio17 of at least 75. The following three plots of current and two future climate scenarios show a northward expansion of suitable habitat from western Nepal and Uttarakhand of India. Because bio17 is not the only important predictor of KMD distribution and because the actual impact of bio17 on the local distribution of KMD cannot be accurately predicted by marginal response curve, we do not expect a perfect match between the distribution of bio17>75 and KMD probability.


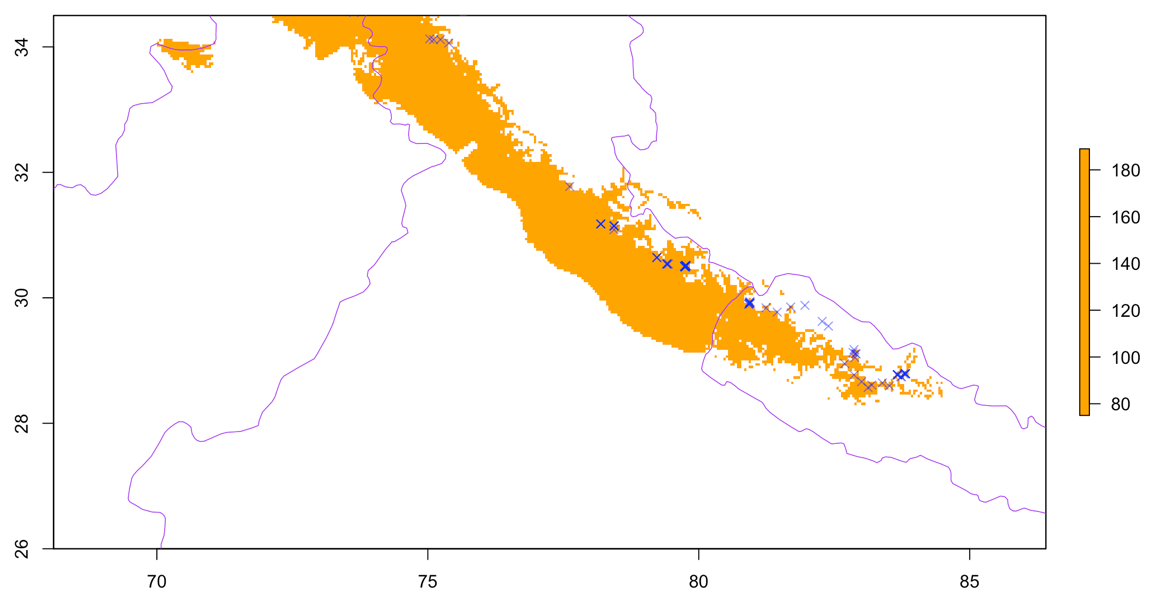


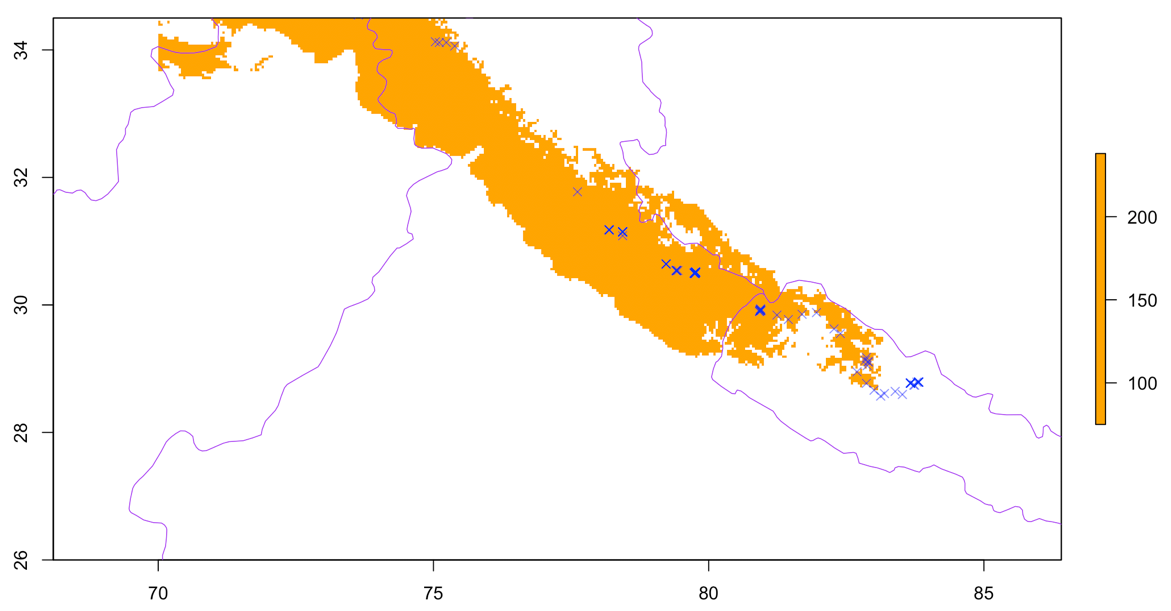


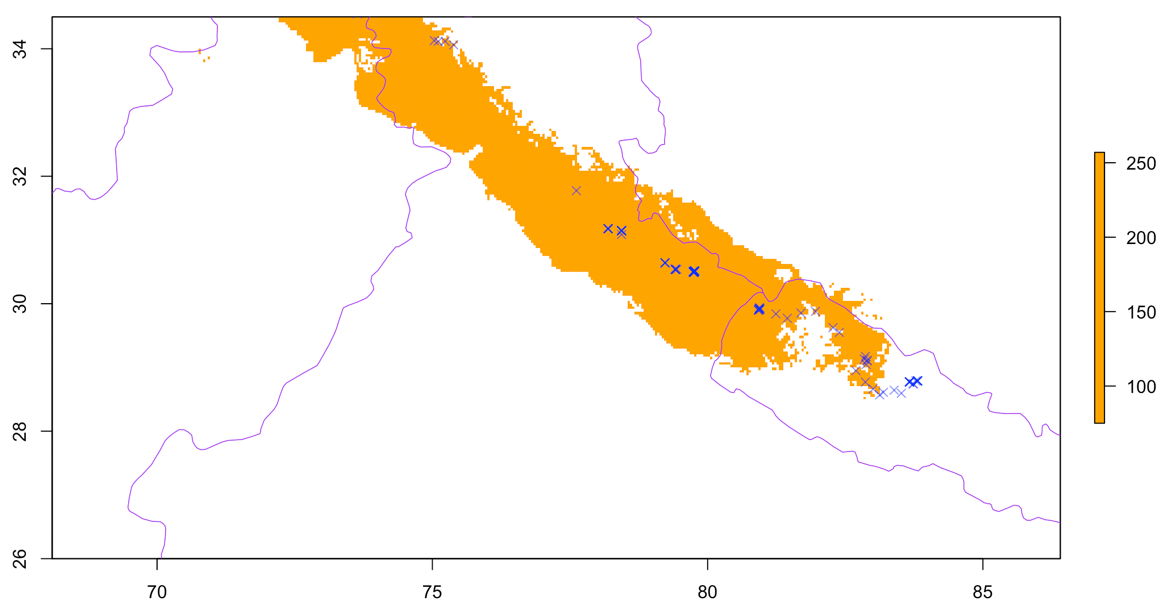


Northward expansion of suitable range of bio17 for KMD. Pixels with bio17 of 75 or more are shown in the plots. From the current spatial coverage of bio17>75 (top panel), the future spatial coverage of bio17>75 in 2050 expands to the north under bc26 (middle panel) and bc45 (bottom panel). Because the relationship between bio17 and probability is not linear, to show the spatial shift of suitable habitat, we have shown only one color for raster cells >=75.
