# Supplementary material for: Projected distribution and climate refugia of endangered Kashmir musk deer Moschus cupreus in greater Himalaya, South Asia
Source: Sci Rep. 2020 Jan 30;10:1511. doi: 10.1038/s41598-020-58111-6 (PMC6992763; doi:10.1038/s41598-020-58111-6)
Supplement: Supplementary file 2 — Supplementary Figures. [file 41598_2020_58111_MOESM2_ESM.doc]

# Projected distribution and climate refugia of endangered Kashmir musk deer *Moschus cupreus* in greater Himalaya, South Asia

Paras Bikram Singh ^1,6^, Kumar Mainali ^2^, Zhigang Jiang^3, 4^, Arjun Thapa ^5^, Naresh Subedi ^6^, Mahammad Naeem Awan ^7^, Orus Ilyas ^8^, Himal Luitel^9^, Zhixin Zhou^1^, Huijian Hu ^1*^

***^1^*** *Guangdong Key Laboratory of Animal Conservation and Resource Utilization, Guangdong Institute of Applied Biological Resources, Xin’ganxi Road, Guangzhou, China,* ***^2^****National Socio-Environmental Synthesis Center, Annapolis, Maryland, USA,* ***^3^****Key Laboratory of Animal Ecology and Conservation Biology, Institute of Zoology, Chinese Academy of Sciences, Beichen West Road, Beijing 100101, China,* ***^4^****University of Chinese Academy of Science, Beijing 100049, China, , ^5^Small Mammals Conservation and Research Foundation, Kathmandu, Nepal, ^6^* *National Trust for Nature Conservation, Khumaltar, Lalitpur, Nepal, ^7^Earth Day Network, Islamabad, Pakistan, ^8^Department of Wildlife Sciences, Aligarh Muslim University, Aligarh, India, ^9^Center of Biotechnology, Agriculture and Forestry University, Rampur, Chitwan, Nepal.*

*Corresponding author, email:13922339577@139.com

**List of the supplementary figures**

**Supplementary Fig. S1.** Categorical habitat suitability of KMD. The continuous surface of probability was divided into four categories of habitat suitability as below: unsuitable (0–0.2), marginally suitable (0.2–0.5), suitable (0.5–0.7), and highly suitable (0.7–1.0). The probability surface was generated by species distribution models built with Maximum Entropy (MaxEnt) Models. The map was plotted using R 3.4.3 (R Foundation for Statistical Computing, Vienna, Austria, http://www.r-project.org/)

**Supplementary Figs. S2.** The prediction was made for the entire Himalaya (see Figure 3.1) but we only show a part of it that includes all sites with any kind of suitable habitat. The parts of the Himalaya not shown have only unsuitable habitat. (a) RCP 2.6 climate scenario in 2050s, (b) RCP 2.6 climate scenario in 2070s, (c) RCP 4.5 in 2050s, (d) RCP 4.5 in 2070s, (e) RCP 6.0 in 2050s (f) RCP 6.0 in 2070s, (g) RCP 8.5 in 2050s and (h) RCP 8.5 in 2070s. The all maps were plotted using R 3.4.3 (R Foundation for Statistical Computing, Vienna, Austria, [*http://www.r-project.org/)*](http://www.r-project.org/))

**
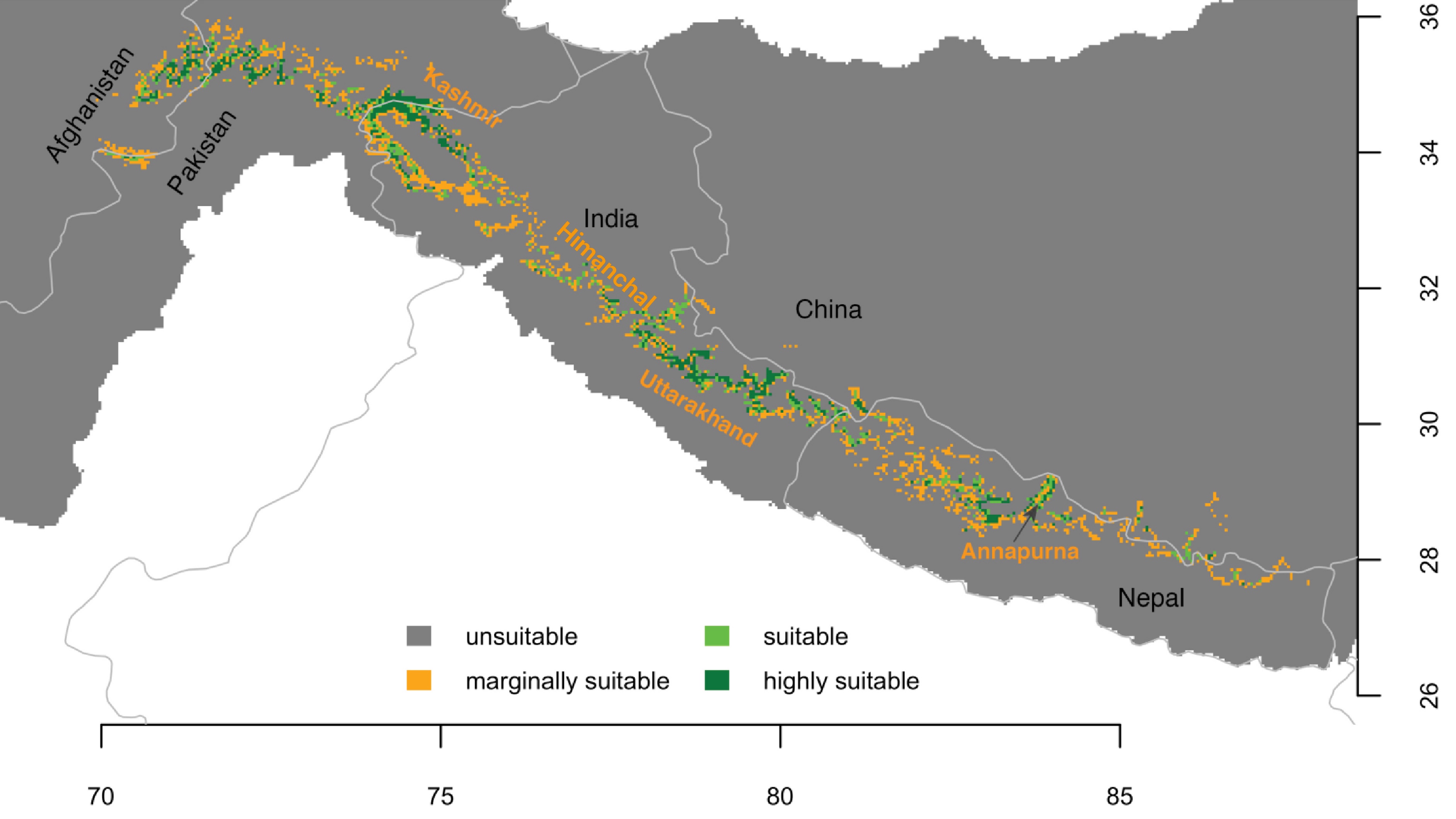
**

**Supplementary Fig. S1.** Categorical habitat suitability of KMD. The continuous surface of probability was divided into four categories of habitat suitability as below: unsuitable (0–0.2), marginally suitable (0.2–0.5), suitable (0.5–0.7), and highly suitable (0.7–1.0). The probability surface was generated by species distribution models built with Maximum Entropy (MaxEnt) Models. The map was plotted using R 3.4.3 (R Foundation for Statistical Computing, Vienna, Austria, http://www.r-project.org/)


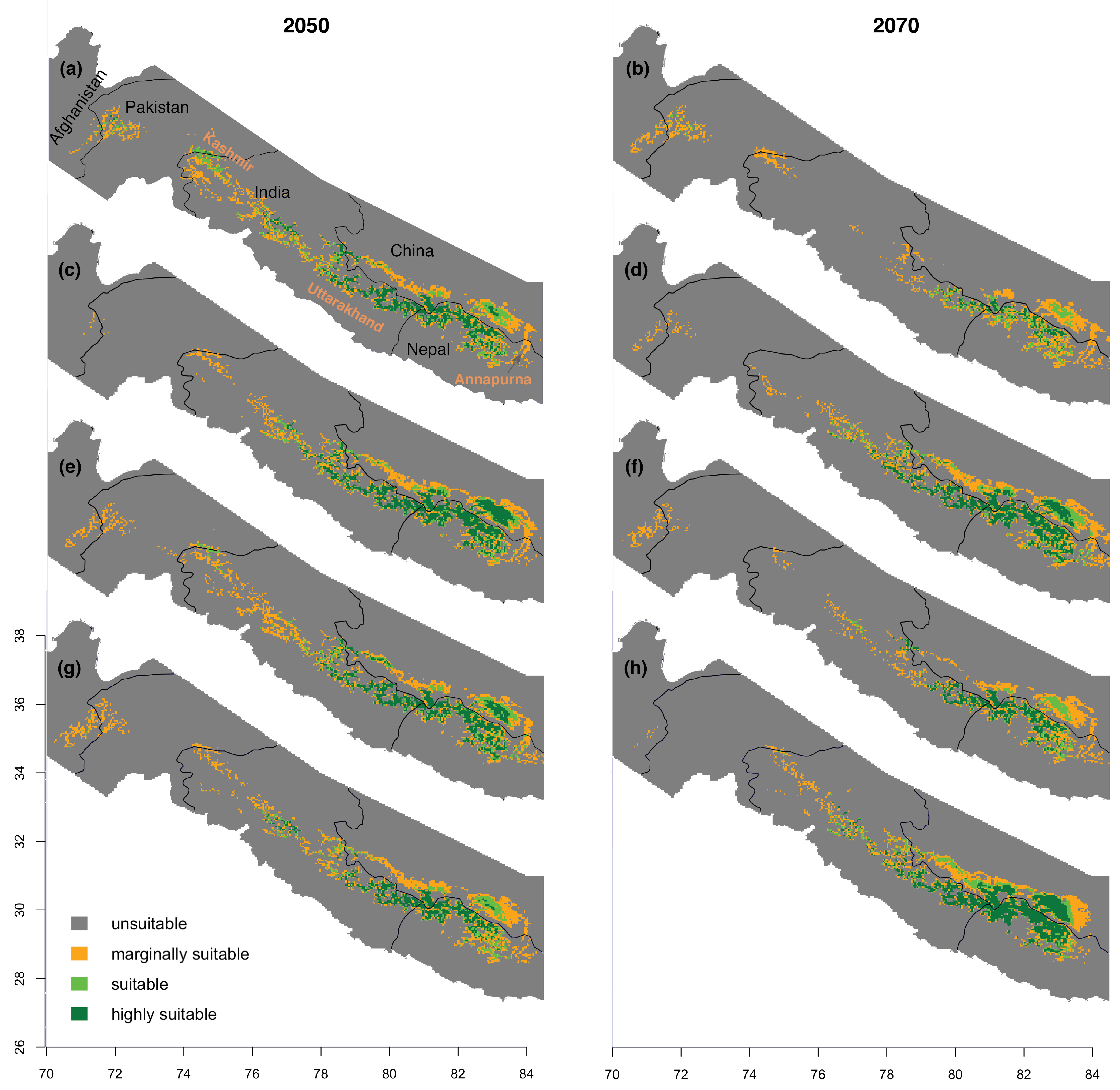


**Supplementary Figs. S2.** The prediction was made for the entire Himalaya (see Fig. 3) but we only show a part of it that includes all sites with any kind of suitable habitat. The parts of the Himalaya not shown have only unsuitable habitat. (a) RCP 2.6 climate scenario in 2050s, (b) RCP 2.6 climate scenario in 2070s, (c) RCP 4.5 in 2050s, (d) RCP 4.5 in 2070s, (e) RCP 6.0 in 2050s (f) RCP 6.0 in 2070s, (g) RCP 8.5 in 2050s and (h) RCP 8.5 in 2070s. All maps were plotted using R 3.4.3 (R Foundation for Statistical Computing, Vienna, Austria, http://www.r-project.org/)
